# Oxidative Stress as a Main Contributor of Retinal Degenerative Diseases

**DOI:** 10.3390/antiox11061190

**Published:** 2022-06-17

**Authors:** Isabel Pinilla, Victoria Maneu

**Affiliations:** 1 Aragon Institute for Health Research (IIS Aragon), 50009 Zaragoza, Spain; 2 Department of Ophthalmology, Lozano Blesa, University Hospital, 50009 Zaragoza, Spain; 3 Department of Surgery, University of Zaragoza, 50009 Zaragoza, Spain; 4 Department of Optics, Pharmacology and Anatomy, University of Alicante, 03690 San Vicente del Raspeig, Spain; vmaneu@ua.es

Retinal degenerative diseases, including inherited retinal dystrophies (IRDs) and acquired multifactorial diseases, such as age-related macular degeneration (AMD), diabetic retinopathy (DR) or ganglion cell damage secondary to glaucoma or other pathologies, are the main causes of blindness in developed countries [1,2]. These different disease entities have a similar end, that is, the irreversible loss of visual function. Although their aetiology is different, they share common mechanisms that drive the death of retinal neurons. The main keys to retinal damage and cell death are inflammation and oxidative stress [3].

The retina is exposed to high levels of light and oxidative stress. It is one of the most metabolically active tissues in an organism, with photoreceptors being one of the largest oxygen consumers in the central nervous system, mainly due to the large accumulation of mitochondria in the ellipsoid. A large energy supply is needed for correct visual function [4]. Reactive oxygen and nitrogen species (RNOS) are produced as a regular part of retinal metabolism. Maintaining low and controlled levels of RNOS allows the control of cell signalling processes by reversible oxidative and nitrosative modifications of key redox-sensitive residues in regulatory proteins such as MAPK or PI3K/Akt. Redox modifications also regulate gene expression, transcription factors and epigenetic pathways [5]. Changes in the expression of some genes can also modify the oxidative stress effects [6]. Under physiological conditions, there is a cellular balance between RNOS formation and removal as the organism has its own defence system to neutralise or catalyse RNOS and to repair damage from enzymatic antioxidants such as copper–zinc and manganese superoxide dismutases, catalase, peroxiredoxin, glutathione peroxidase, and glutathione reductase. Other nonenzymatic antioxidants, such as vitamins E, A and C, are also involved in the protection of intracellular components against RNOS. This type of protection can also be realized by natural compounds, such as flavonoids, carotenoids or curcumin, which can be found in a diet containing dairy products [7]. When the balance between RNOS formation and its removal is disrupted, oxidative stress arises, promoting the oxidation of proteins, lipids and DNA and activating inflammation and cell death pathways that lead to retinal degeneration. Photoreceptors are extremely sensitive to high ROS levels and lipid peroxidation due to the large surface area of membranes enriched with polyunsaturated fats.

Numerous data show that oxidative stress has a main role in retinal degenerative diseases such as AMD, glaucoma and retinitis pigmentosa (RP) [8,9]. From this perspective, the administration of antioxidant drugs and dietary supplements with antioxidant compounds have shown good results in both animal models and clinical trials [10,11]. It must be noted that in the translation of the results from animal research into clinical research, there are many challenges, including multifactorial diseases and/or multiple genetic and phenotypic variations. Hence, in most cases, there are no good animal models available to reliably model human disease. Increasing evidence also points to the potential benefits of combined antioxidant therapy. Several compounds with antioxidant, anti-inflammatory and antiapoptotic effects have been or are being tested in clinical trials for the treatment of IRDs, including RP, Usher syndrome and Stargardt disease and as therapeutic approaches to DR and AMD. However, thus far, clinical trials are still too heterogeneous with respect to the molecules analysed as well as the dosage and results. Another problem in obtaining results is that IRDs are rare diseases. Therefore, it is difficult to recruit enough patients to achieve the expected result. An exhaustive analysis of data and additional studies, including long-term assays, are needed to establish the safety and efficacy of these compounds.

Neuroinflammation and cell death are always present in retinal neurodegenerative diseases, in which multiple inflammatory mediators trigger, worsen or perpetuate the degenerative process that ends in cell death. Hence, until cell- and gene-based therapies evolve enough to allow us to prevent, stop or revert degeneration, the use of antioxidant, anti-inflammatory and antiapoptotic therapies, most likely in combination, is possibly one of our best attempts to slow the degenerative process and preserve visual function for some time. As a part of this attempt, the development of new retinal delivery systems will also greatly improve the chances of using topical administration as a useful route for molecules to reach the retina, avoiding systemic administration or intravitreal injections. Nanocarriers or cell encapsulating technology are examples of this case in a field that is continuously evolving.

In the near future, gene-based therapies combined with cell replacement and the use of optogenetics, together with the use of antioxidants and anti-inflammatory drugs, will provide effective therapeutic weapons for retinal degenerative diseases. “The best is yet to come”.
